# Phase 1 study of the MDM2 antagonist RO6839921 in patients with acute myeloid leukemia

**DOI:** 10.1007/s10637-020-00907-4

**Published:** 2020-02-04

**Authors:** Geoffrey L. Uy, Sarit Assouline, Anne-Marie Young, Steven Blotner, Brian Higgins, Lin-Chi Chen, Karen Yee

**Affiliations:** 1 Division of Oncology, Washington University School of Medicine, St Louis, MO USA; 2 Jewish General Hospital, Montreal, QC Canada; 3 Pharma Research and Early Development, Roche Innovation Center, Welwyn Garden City, UK; 4 Pharma Research and Early Development, Roche Innovation Center, New York, NY USA; 5 Princess Margaret Cancer Centre, Toronto, ON Canada

**Keywords:** Acute myeloid leukemia, MDM2, Idasanutlin, Safety

## Abstract

In acute myeloid leukemia (AML), *TP53* mutations and dysregulation of wild-type p53 is common and supports an MDM2 antagonist as a therapy. RO6839921 is an inactive pegylated prodrug of the oral MDM2 antagonist idasanutlin (active principle [AP]) that allows for IV administration. This phase 1 monotherapy study evaluated the safety, pharmacokinetics, and pharmacodynamics of RO6839921 in patients with AML. Primary objectives identified dose-limiting toxicities (DLTs) and maximum tolerated dose (MTD). Secondary objectives assessed pharmacokinetic, pharmacodynamic, and antileukemic activity. A total of 26 patients received 120–300 mg AP of idasanutlin. The MTD was 200 mg, with DLTs at 250 (2/8 patients) and 300 mg (2/5). Treatment–related adverse events in >20% of patients were diarrhea, nausea, vomiting, decreased appetite, and fatigue. Six deaths (23.1%) occurred, all unrelated to treatment. Pharmacokinetics showed rapid and near-complete conversion of the prodrug to AP and dose-proportional exposure across doses. Variability ranged from 30%–47% (22%–54% for idasanutlin). *TP53* was 21 (87.5%) wild-type and 3 mutant (12.5%). The composite response rate (complete remission [CR], CR with incomplete hematologic recovery/morphological leukemia-free state [CRi/MLFS], or CR without platelet recovery [CRp]) was 7.7%. Antileukemic activity (CR, CRi/MLFS, partial response, hematologic improvement/stable disease) was observed in 11 patients (disease control rate, 42%): 10/11 were *TP53* wild-type; 1 had no sample. p53 activation was demonstrated by MIC-1 induction and was associated with AP exposure. There was not sufficient differentiation or improvement in the biologic or safety profile compared with oral idasanutlin to support continued development of RO6839921. NCT02098967.

## Introduction

The p53 protein is a growth-suppressive and pro-apoptotic protein that plays a central role in the protection of cells from tumor development. [1, 2] In normal cells, a close relationship exists between p53 and its primary regulator, murine double minute 2 (MDM2), which controls both p53 expression and degradation. MDM2 regulates p53 through a negative feedback loop. When nuclear p53 levels are elevated, they activate the transcription of the *MDM2* gene; this allows MDM2 to bind to p53, blocking its transactivation domain and targeting p53 for ubiquitin-dependent degradation. [1–3]

The p53 signaling pathway is frequently inactivated in acute myeloid leukemia (AML). The *TP53* mutation rate is <10% of cases of de novo AML [4]; however, inactivation of wild-type p53 occurs in many patients with AML by alternative mechanisms, including overexpression of MDM2, in order to allow proliferation and leukemogenesis. [5] Therefore, treatment with an MDM2 antagonist is a therapeutic option to restore p53 activity in these cases. [1, 6] MDM2 antagonists block p53-MDM2 binding, stabilize p53, and activate p53 signaling, thereby inducing cell cycle arrest and apoptosis. Idasanutlin, an oral MDM2 antagonist of the nutlin family of compounds, [6, 7] is being evaluated in phase 1 to 3 clinical trials in patients with solid and hematologic malignancies. [8, 9] In clinical trials in patients with AML who received MDM2 antagonists, MDM2 gene expression was related to clinical response. [10–12]

RO6839921 is an inactive pegylated prodrug of idasanutlin that allows for the solubility needed for IV administration, with the goals of improving exposure variability and pharmacokinetic parameters, reducing gastrointestinal toxicity in the absence of prophylaxis, and potentially improving efficacy compared with oral idasanutlin. The active principle (AP; idasanutlin) is released upon cleavage of its pegylated tail by esterases in the blood. IV-administered RO6839921 showed antitumor activity at nontoxic doses in established osteosarcoma and AML xenograft models in immunocompromised mice. [13] These nonclinical pharmacology results supported further evaluation of RO6839921 in clinical studies. This phase 1 study evaluated the safety, tolerability, pharmacokinetics, and pharmacodynamics of RO6839921 in patients with AML.

## Methods

### Patients

This phase 1 study (NCT02098967) was an open-label, first-in-human, multicenter, dose-escalation study of RO6839921 in patients with solid tumors and in patients with AML; results for solid tumors will be reported independently. Patients aged ≥18 years with relapsed/refractory AML, untreated AML with antecedent hematologic disorder, or high-risk de novo AML as defined by the 2010 European LeukemiaNet (ELN) criteria with Eastern Cooperative Oncology Group performance status of ≤2 were eligible. [14] Patients with central nervous system leukemia or any severe and/or uncontrolled medical conditions or other conditions that could affect their participation were excluded. The study was conducted in accordance with the principles of the International Conference on Harmonization Good Clinical Practice guidelines. All procedures performed in studies involving human participants were in accordance with the ethical standards of the institutional and/or national research committees at the study sites and with the 1964 Declaration of Helsinki and its later amendments or comparable ethical standards. Informed consent was obtained from all participants included in the study.

### Study design

RO6839921 was administered as an IV infusion over approximately 1 h once daily for 5 days every 28 days. Dose escalation was performed using a modified rolling 6 design initiated at or below the dose at which grade ≥ 2 hematologic toxicity or projected efficacious exposure was reached in the solid tumor arm (120 mg AP). [15] Based on this design, the doses tested were 120, 200, 250, and 300 mg (in mg of AP).

The primary objectives of the study were to determine the maximum tolerated dose (MTD) and recommended phase 2 dose of RO6839921 and to characterize dose-limiting toxicities (DLTs) and the overall safety profile. The secondary objectives were to determine the pharmacokinetic parameters of RO6839921 and the AP and to assess the pharmacodynamic effects of RO6839921 and clinical responses. Treatment continued until disease progression, unacceptable toxicity, withdrawal of consent, or investigator discretion.

### Assessments and analysis

Patients receiving ≥1 dose of RO6839921 were considered evaluable for safety. Adverse events (AEs) were graded according to the National Cancer Institute Common Terminology Criteria for Adverse Events (version 4.03).

The DLT-evaluable population was defined as all patients who received ≥80% of study medication and completed the first 28-day treatment cycle. In addition, patients who had a DLT but did not meet the minimum dosing requirements were considered evaluable for DLTs. DLTs were assessed during the first treatment cycle (28 days) and included prolonged grade 4 neutropenia and prolonged grade 3/4 thrombocytopenia lasting ≥42 days from the start of the cycle in the absence of evidence of active AML as well as clinically significant grade 3 to 5 nonhematologic toxicity. The MTD was defined as the highest dose level tested with 0 to 1 DLT in a cohort of 6.

Plasma pharmacokinetic assessments of RO6839921 and AP concentrations were conducted in all patients during the first cycle of treatment on the first and fifth days of dosing immediately before dosing and at multiple postdose time points using a validated liquid chromatography–tandem mass spectrometry method, with pharmacokinetic parameters estimated using standard noncompartmental methods. Assessment of macrophage inhibitory cytokine 1 (MIC-1) protein levels was measured in serum before and after administration of RO6839921 using an enzyme-linked immunosorbent assay. *TP53* mutation status was measured by next-generation sequencing at baseline.

The efficacy population was defined as all patients who received ≥80% of study medication and completed the first 28-day treatment cycle. Efficacy was evaluated on day 1 of every cycle starting with cycle 2. A composite response rate was calculated by determining the number of patients who achieved a best response (complete remission [CR], CR without platelet recovery [CRp], or CR with incomplete recovery of peripheral counts [CRi]/morphological leukemia-free state [MLFS]) divided by the total number of patients in the efficacy population. Additional outcomes were partial response with ≥50% decrease in bone marrow blasts, hematologic improvement measures, and disease progression. Hematologic improvement/stable disease (HI/SD) was defined as decreased peripheral blast percentage, decreased frequency of transfusions, and/or improvement in peripheral cell counts in the absence of CR in the marrow and were considered by Investigators and Sponsor on a case by case basis for continuation of treatment.

## Results

### Patient disposition and characteristics

This study was conducted at 6 sites in the United States and Canada between April 2014 and May 2018. A total of 26 patients with AML were treated at 4 doses: 120 mg AP (*n* = 6), 200 mg AP (*n* = 7), 250 mg AP (*n* = 8), and 300 mg AP (*n* = 5). Patients received a median of 5 doses (range, 4–10). The median treatment duration was 5 days (range, 4–86 days), and the median cumulative dose was 1225 mg (range, 600–3000 mg). Treatment was discontinued in 10 patients (38.5%) due to progression of disease, 9 patients (34.6%) due to physician decision (perceived lack or loss of clinical benefit), and 7 patients (26.9%) due to AEs.

The median age was 65 years (Table 1). The majority of patients had an ELN intermediate 2 [14] or adverse risk and had an Eastern Cooperative Oncology Group performance status of 0 or 1. Half the patients had prior cancer, and 81% had wild-type *TP53* status.

**Table 1:** Baseline demographic and clinical characteristics

Characteristics	120 mg AP (*n* = 6)	200 mg AP (*n* = 7)	250 mg AP (*n* = 8)	300 mg AP (*n* = 5)	Total (*N* = 26)
Male, n (%)	3 (50.0)	5 (71.4)	3 (37.5)	0	11 (42.3)
Median age (range), years	50 (24–73)	58 (47–74)	74 (64–80)	49 (33–71)	65 (24–80)
ECOG PS, n (%)					
0	4 (67)	2 (29)	1 (13)	1 (20)	8 (31)
1	2 (33)	5 (71)	7 (88)	4 (80)	18 (69)
ELN risk at diagnosis, n (%)					
Favorable	0	1 (14)	1 (13)	0	2 (8)
Intermediate 1	0	3 (43)	3 (38)	1 (20)	7 (27)
Intermediate 2	1 (17)	2 (29)	1 (13)	1 (20)	5 (19)
Adverse	5 (83)	1 (14)	3 (38)	3 (60)	12 (46)
Antecedent hematologic disorder, n (%)*	1 (17)	0	3 (38)	2 (40)	6 (23)
Prior cancer, n (%)^†^	3 (50)	5 (71)	2 (25)	3 (60)	13 (50)
No. of prior regimens, n (%)					
0	1 (16.7)	0	2 (25)	1 (20)	4 (15.4)
1	1 (16.7)	2 (28.6)	3 (37.5)	1 (20)	7 (26.9)
2	2 (33.3)	2 (28.6)	3 (37.5)	2 (40)	9 (34.6)
3	1 (16.7)	2 (28.6)	0	1 (20)	4 (15.4)
4	1 (16.7)	1 (14.3)	0	0	2 (7.7)
Prior allogeneic transplant, n (%)	1 (17)	4 (57)	1 (13)	1 (20)	7 (27)
Response to prior therapy^‡^					
No prior therapy	1 (17)	0	2 (25)	1 (20)	4 (15)
Refractory	2 (33)	2 (29)	3 (38)	3 (60)	10 (38)
CR1 < 3 months	1 (17)	1 (14)	0	0	2 (8)
CR1 3– 12 months	2 (33)	4 (57)	2 (25)	1 (20)	9 (35)
CR1 > 12 months	0	0	1 (13)	0	1 (13)
*TP53* status					
Not evaluable	1 (17)	1 (14)	0	0	2 (8)
Evaluable	5 (83)	6 (86)	8 (100)	5 (100)	24 (92)
Wild type	5 (100)	5 (83)	8 (100)	3 (60)	21 (81)
Mutant	0	1 (17)	0	2 (40)	3 (12)

*AP* active principle, *CR1* complete remission with first treatment received, *ECOG PS* Eastern Cooperative Oncology Group performance status, *ELN* European LeukemiaNet

*Includes chronic myelomonocytic leukemia, myelodysplastic syndromes, and essential thrombocythemia

^†^Prior cancer includes lymphoma (*n* = 4), prostate cancer, and breast cancer

^‡^A first complete remission/complete remission without platelet recovery <12 months is associated with poor response rates in relapse

### DLT and MTD determination

All 26 patients were evaluable for DLTs. Four patients (15.4%) experienced DLTs: 2 in the 300-mg cohort experienced 2 DLTs (colitis [grade 3, serious] and electrocardiogram QT interval prolonged [grade 4, serious]) and 2 in the 250-mg cohort experienced 2 DLTs (diarrhea [grade 3] and stomatitis [grade 3]). The MTD (defined as the highest dose level tested with 0–1 DLT in a cohort of 6 patients) in patients with AML was 200 mg AP (0 DLTs in 7 patients).

### Safety

All 26 patients received ≥1 dose of RO6839921 and were therefore considered safety evaluable. The most common AEs were nausea (57.7%), decreased appetite and febrile neutropenia (53.8% each), diarrhea and hypomagnesemia (50.0% each), hypokalemia (42.3%), constipation, fatigue, and vomiting (38.5% each), hypotension, peripheral edema, and stomatitis (34.6% each), and abdominal pain, hypophosphatemia, and hyperphosphatemia (30.8% each) (Table 2). All but 1 patient had AEs of grade ≥ 3 (25 patients [96.2%]), and the most common were febrile neutropenia (53.8% of patients), hypokalemia (23.1%), lung infection (15.4%), and hypophosphatemia and stomatitis (11.5% each). Most patients experienced a treatment-related AE (24 patients [92.3%]). The most common treatment-related AEs were diarrhea and nausea (50.0% each), vomiting (34.6%), decreased appetite (30.8%), and fatigue (26.9%) (Table 2). Serious AEs occurred in 22 patients (84.6%) (Table 3). The most common was febrile neutropenia in 13 patients (50.0%).

**Table 2:** Summary of adverse events per MedDRA preferred term (≥ 30% of patients for any adverse events or ≥ 5% treatment-related adverse events)

Patients with an AE, n (%) *N* = 26	All AEs		Treatment-related AEs	
	Any grade	Grade ≥ 3	Any grade	Grade ≥ 3
Nausea	15 (57.7)	0	13 (50.0)	3 (11.5)
Decreased appetite	14 (53.8)	0	8 (30.8)	0
Febrile neutropenia	14 (53.8)	14 (53.8)	3 (11.5)	0
Hypomagnesemia	13 (50.0)	0	1 (3.8)	0
Diarrhea	13 (50.0)	1 (3.8)	13 (50.0)	1 (3.8)
Hypokalemia	11 (42.3)	6 (23.1)	0	0
Vomiting	10 (38.5)	0	9 (34.6)	0
Constipation	10 (38.5)	0	0	0
Fatigue	10 (38.5)	1 (3.8)	7 (26.9)	1 (3.8)
Stomatitis	9 (34.6)	3 (11.5)	5 (19.2)	1 (3.8)
Hypotension	9 (34.6)	0	0	0
Edema peripheral	9 (34.6)	0	1 (3.8)	0
Hypophosphatemia	8 (30.8)	3 (11.5)	0	0
Hyperphosphatemia	8 (30.8)	0	0	0
Abdominal pain	8 (30.8)	1 (3.8)	5 (19.2)	0
Epistaxis	5 (19.2)	2 (7.7)	3 (11.5)	2 (7.7)
Dyspepsia	4 (15.4)	0	2 (7.7)	0
Alopecia	4 (15.4)	0	4 (15.4)	0

*AE* adverse event, *MedDRA* Medical Dictionary for Regulatory Activities

**Table 3:** Summary of serious adverse events (per MedDRA preferred term)

Patients with an SAE, n (%) *N* = 26	All AEs	Study drug–related AEs
Febrile neutropenia	13 (50.0)	2 (7.7)
Lung infection	3 (11.5)	0
Aspergillus infection	1 (3.8)	0
Bacterial sepsis	1 (3.8)	0
Cellulitis	1 (3.8)	0
*Enterobacter* bacteremia	1 (3.8)	0
*Enterobacter* infection	1 (3.8)	0
Enterococcal sepsis	1 (3.8)	0
Proctitis herpes	1 (3.8)	0
Pseudomonal bacteremia	1 (3.8)	0
Sepsis	1 (3.8)	0
Colitis	1 (3.8)	1 (3.8)
Gastritis	1 (3.8)	1 (3.8)
Acute coronary syndrome	1 (3.8)	0
Acute cardiac failure	1 (3.8)	0
Myocardial ischemia	1 (3.8)	0
Blood creatinine increased	1 (3.8)	1 (3.8)
Electrocardiogram QT interval prolonged	1 (3.8)	1 (3.8)
Dyspnea	1 (3.8)	0
Pyrexia	1 (3.8)	0
Menorrhagia	1 (3.8)	0
Thrombosis	1 (3.8)	0

*AE* adverse event, *MedDRA* Medical Dictionary for Regulatory Activities, *SAE* serious adverse event

Seven patients (26.9%) experienced 8 AEs leading to withdrawal of study treatment (3 patients each in the 250- and 300-mg cohorts, 1 patient in the 200-mg cohort). One patient (250-mg cohort) reported 2 AEs of acute cardiac failure and sepsis; all other AEs (acute coronary syndrome, acute cardiac failure, bacterial sepsis, diarrhea, and pyrexia) were experienced by 1 patient each. Five patients (19.2%) experienced dose interruptions: 2 in the 120-mg cohort due to febrile neutropenia and aspergillus infection, respectively, both grade 3; 2 in the 200-mg cohort due to febrile neutropenia (1 patient had 1 febrile neutropenia event and 1 patient had 2 febrile neutropenia events); and 1 in the 250-mg cohort due to increased blood creatinine and decreased renal creatinine clearance, both grade 2.

Six deaths (23.1%) were reported during the study. Four deaths were due to progressive disease or relapse. Two deaths resulted from AEs with a fatal outcome (acute coronary syndrome and bacterial sepsis); both of these were judged to be unrelated to the study medication.

### Pharmacokinetics

**Fig. 1:**
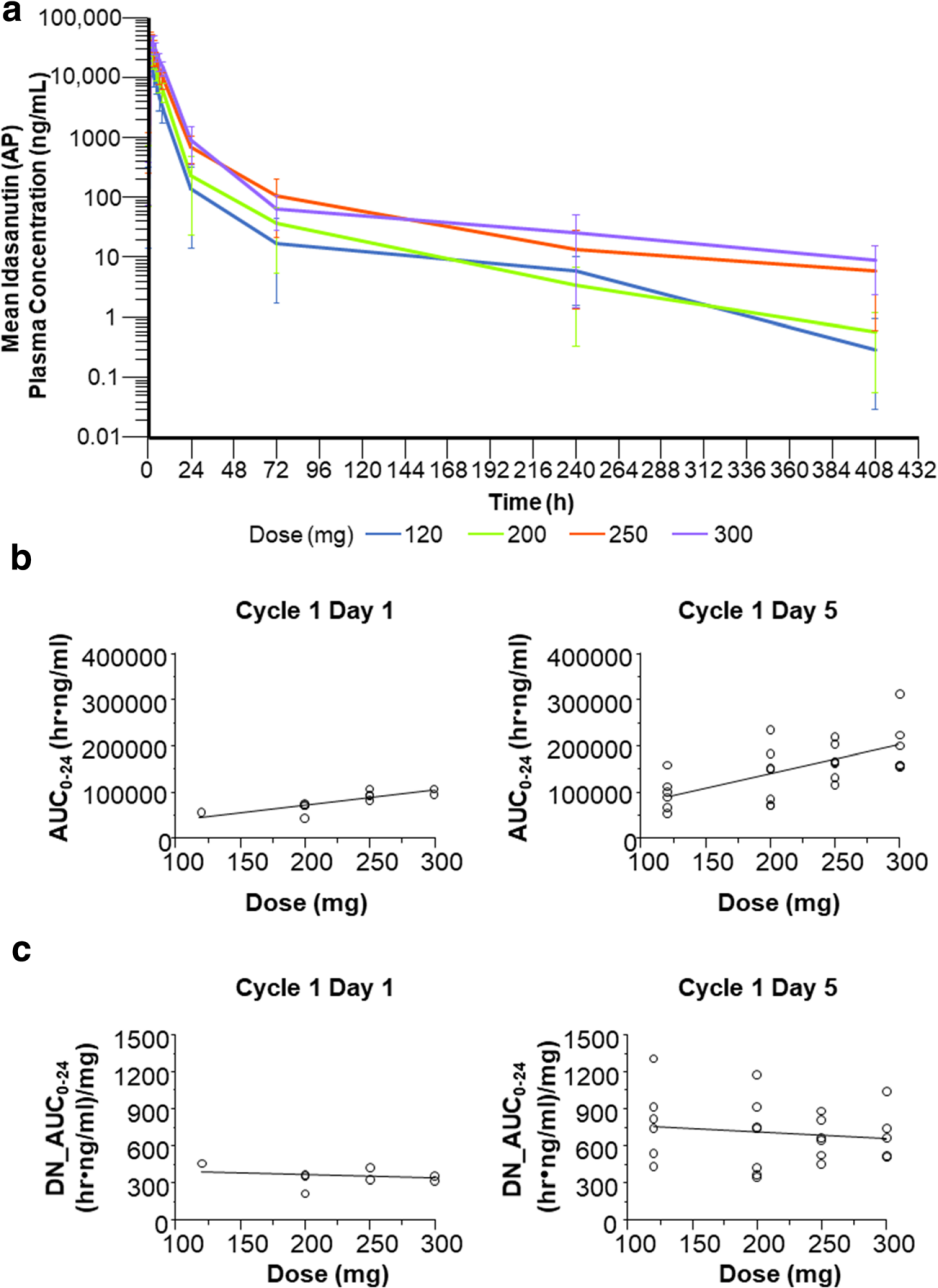
**Pharmacokinetic analyses. a** Mean plasma idasanutlin (AP) concentration-time profiles following RO6839921 (prodrug) administration; **b** AP dose-exposure relationship on days 1 and 5 for absolute AUC; **c** AP dose-exposure relationship on days 1 and 5 for dose-normalized AUC. AP, active principle; DN, dose-normalized; AUC_0-24_, 24-h area under the plasma concentration-time curve

Mean plasma prodrug concentration-time profiles following RO6839921 administration of the AP are presented in Fig. 1a; pharmacokinetic parameters for RO6839921 and the AP are summarized in Tables 4 and 5. The dose-exposure relationship for the AP on days 1 and 5 was approximately linear and dose proportional (Fig. 1b and c). For the 200-, 250-, and 300-mg cohorts, the mean 24-h area under the plasma concentration-time curve on day 5 was above the target exposure of 100 h•μg/mL for RO6839921 (Table 4) and the AP (Table 5) based on preclinical studies. [13]

**Table 4:** Pharmacokinetic parameters for RO6839921 (prodrug)

Dose, mg		Day 1			Day 5				Ratio (day 5/day 1)	
		t_max_, h	C_max_, ng/mL	AUC_0–24_, h•ng/mL	t_max_, h	C_max_, ng/mL	AUC_0–24_, h•ng/mL	t_1/2_, h*	C_max_, ng/mL	AUC_0–24_, h•ng/mL
120	N	6	6	6	6	6	6	6	6	6
	Mean (SD)^†^	1.05 (1.00–2.03)	19,100 (4970)	86,200 (27,700)	1.03 (1.00–1.18)	18,000 (5530)	89,600 (30,000)	23.0 (26.0)	0.944 (0.102)	1.04 (0.10)
	CV%	33.6	26.1	32.1	6.7	30.7	33.4	113	10.8	9.9
200	N	7	7	7	7	7	7	7	7	7
	Mean (SD)^†^	1.12 (1.00–1.98)	27,000 (4070)	126,000 (38,400)	1.03 (1.00–2.08)	32,400 (13,400)	164,000 (40,700)	20.8 (9.85)	1.25 (0.724)	1.39 (0.46)
	CV%	28.7	15.1	30.5	33.4	41.4	24.7	47.5	57.7	33.1
250	N	8	8	8	6	6	6	6	6	6
	Mean (SD)^†^	1.08 (1.02–1.92)	76,500 (84,400)	241,000 (85,400)	1.05 (1.00–2.00)	43,500 (12,500)	258,000 (79,500)	50.0 (28.4)	0.832 (0.368)	1.14 (0.37)
	CV%	26.2	110	35.4	36.1	28.8	30.9	56.9	44.3	32.0
300	N	5	5	5	5	5	5	5	5	5
	Mean (SD)^†^	2.00 (1.03–2.08)	66,700 (46,900)	304,000 (89,500)	1.98 (1.02–2.00)	41,700 (7910)	335,000 (93,100)	60.3 (49.8)	0.762 (0.252)	1.1 (0.14)
	CV%	33.1	70.4	29.4	28.5	19.0	27.8	82.5	33.1	12.3

AUC_0–24_, 24-h area under the plasma concentration-time curve; C_max_, maximum concentration; CV, coefficient of variation; t_max_, time to maximum concentration

^*^t_½_ (terminal half-life) cannot be determined for day 1 due to a limited sampling schedule

^†^Median (min-max) is given for t_max_

**Table 5:** Pharmacokinetic parameters for the active principle (idasanutlin)

Dose, mg		Day 1			Day 5				Ratio (day 5/day 1)	
		t_max_, h	C_max_, ng/mL	AUC_0–24_, h•ng/mL	t_max_, h	C_max_, ng/mL	AUC_0–24_, h•ng/mL	t_1/2_, h*	C_max_, ng/mL	AUC_0–24_, h•ng/mL
120	N	6	6	1	6	6	6	4	6	6
	Mean (SD)^†^	6.24 (5.92–8.08)	2840 (545)	55,000	4.48 (3.02–8.00)	4920 (1770)	95,300 (37,100)	29.3 (11.3)	1.73 (0.50)	1.76 (0.55)
	CV%	12.4	19.2	–	46.7	36.0	38.9	38.4	29.0	31.1
200	N	7	7	3	7	7	7	6	7	7
	Mean (SD)^†^	5.78 (1.93–7.93)	3540 (1230)	62,100 (16,500)	4.00 (2.98–7.82)	6850 (3000)	135,000 (63,100)	31.2 (9.9)	1.91 (0.52)	2.02 (0.39)
	CV%	39.6	34.9	26.5	38.5	43.8	46.8	31.6	27.1	19.5
250	N	8	8	4	6	6	6	6	6	6
	Mean (SD)^†^	6.77 (3.00–8.60)	4860 (371)	93,000 (9930)	3.08 (2.00–5.95)	8970 (2510)	166,000 (41,000)	26.8 (7.01)	1.82 (0.42)	1.79 (0.30)
	CV%	39.4	7.7	10.7	38.3	28.0	24.7	26.6	23.1	16.8
300	N	5	5	2	5	5	5	5	5	5
	Mean (SD)^†^	6.08 (4.00–7.92)	6120 (1030)	101,000 (7910)	5.92 (1.98–6.02)	11,500 (3610)	209,000 (64,300)	34.3 (7.7)	1.86 (0.33)	1.83 (0.26)
	CV%	24.9	16.8	7.86	42.5	31.4	30.8	22.5	17.9	14.4

AUC_0–24_, 24-h area under the plasma concentration-time curve; C_max_, maximum concentration; CV, coefficient of variation; t_max_, time to maximum concentration

^*^t_½_ (terminal half-life) cannot be determined for day 1 due to a limited sampling schedule

^†^Median (min-max) is given for t_max_

### Biomarkers and pharmacodynamics

**Fig. 2:**
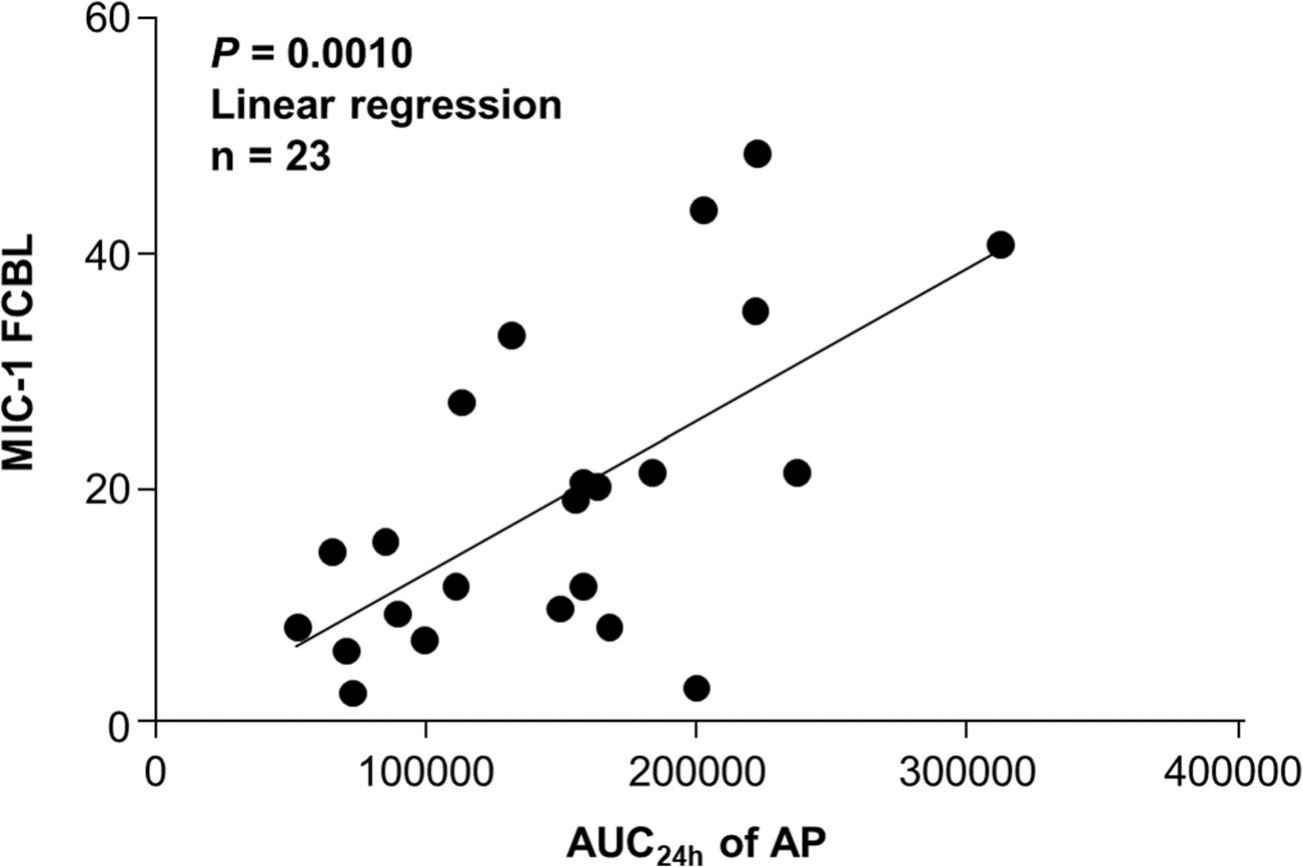
**Pharmacodynamic analyses.** Association of MIC-1 (FCBL) levels with AUC_24h_. AP, active principle; AUC_24h_, 24-h area under the plasma concentration-time curve; FCBL, fold change from baseline; MIC-1, macrophage inhibitory cytokine 1

MIC-1, a secretory protein that is strongly upregulated by activated p53*,* can be detected in the blood of mice bearing human tumor xenografts after treatment with doxorubicin, a genotoxic p53 activator of the MDM2 antagonist nutlin-3. [16] Therefore, MIC-1 could have utility as a progressive disease biomarker for RO6839921. In previous trials that included patients with AML, concentration-related pharmacodynamic biomarker activity of the p53 pathway was demonstrated by increases in MIC-1 levels. [12] In this study, the pharmacodynamic association of change in MIC-1 levels from baseline correlated with steady-state AP exposure (Fig. 2).

Bone marrow samples from 22 patients were evaluated for *TP53* status, and 5 (21%) had ≥1 mutation. One patient had >1 mutation detected. The 2 patients who had a best response of CR/CRi or MLFS did not have *TP53* mutations; however, 1 patient who had HI/SD as a first response had a mutation.

### Efficacy

**Fig. 3:**
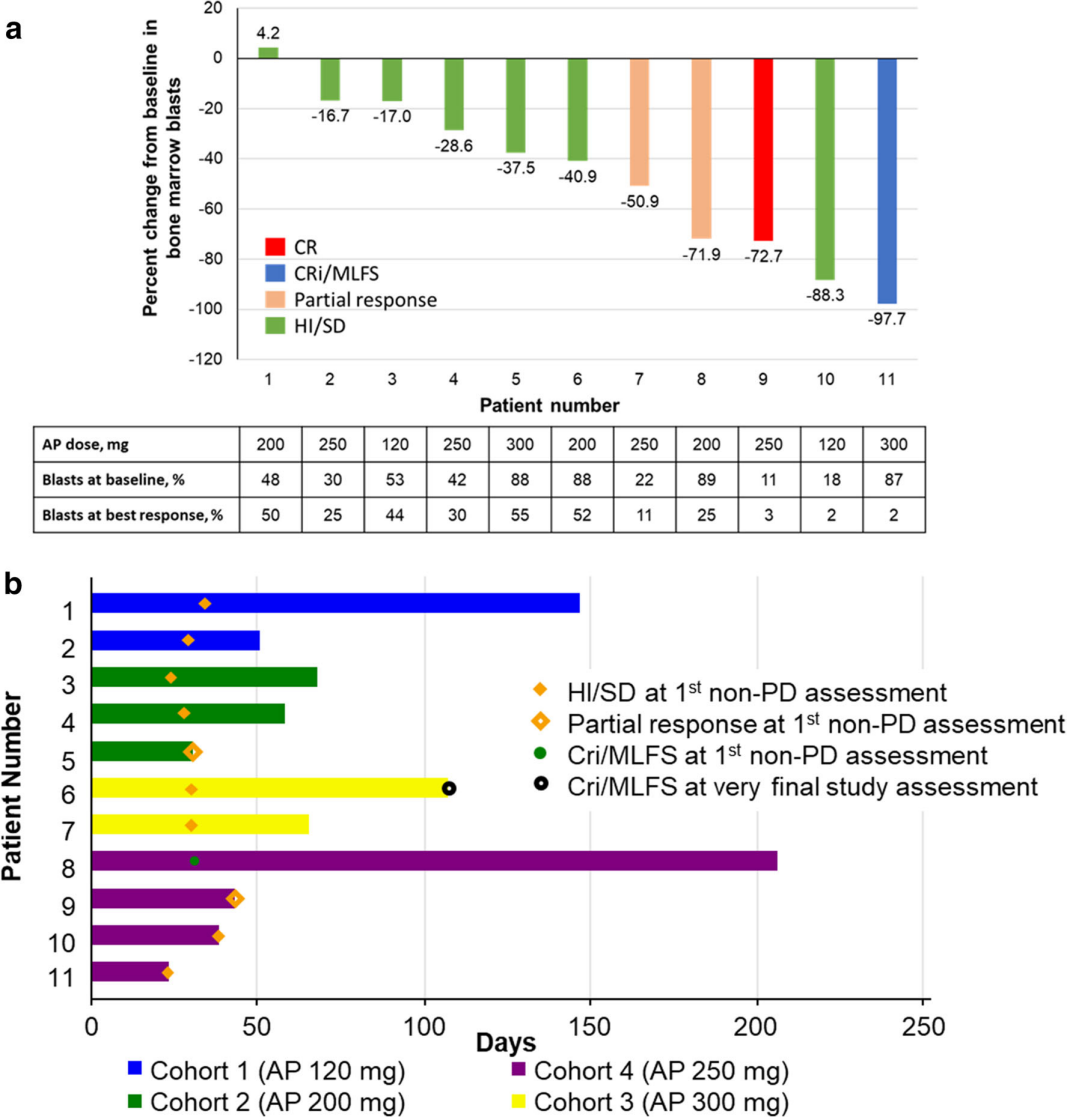
**Antileukemic activity. a** Percent change from baseline in bone marrow blasts at best response in patients with antileukemic activity (CR, CRi/MLFS, PR, or HI/SD). **b** Response duration from treatment start in patients with antileukemic activity (CR, CRi/MLFS, partial response, or HI/SD). Patients without a symbol at the final study assessment experienced relapse. Initial response for patient 6 in part B was CRi/MLFS; best response was CR. AP, active principle; CR, complete remission; CRi, complete remission with incomplete recovery of peripheral counts; HI, hematologic improvement; MLFS, morphological leukemia-free state; PD, progressive disease; SD, stable disease

The composite response rate (CRc; CR, CRp, or CRi/MLFS) was 7.7% (2 patients): 1 patient each in the 250-mg (CR) and 300-mg (CRi/MLFS) cohorts (Table 6). Two patients (7.7%) achieved a partial response: 1 patient each in the 200- and 250-mg cohorts. Seven patients (26.9%) had HI/SD. The disease control rate (CRc, partial response, or HI/SD) was 42% (11 of 26 patients). Five patients were not evaluated: 4 due to AEs and 1 due to physician decision to administer hydroxyurea off protocol because of increasing white count. Of patients who demonstrated antileukemic activity (CR, CRi/MLFS, partial response, or HI/SD), the best change in bone marrow blasts from baseline was varied (Fig. 3a). The median overall duration of antileukemic activity (CR, CRp, CRi/MLFS, partial response, or HI/SD) was 58 days (range, 23–206 days; Fig. 3b).

**Table 6:** Best overall responses

Response, n (%)	120 mg AP (*n* = 6)	200 mg AP (*n* = 7)	250 mg AP (*n* = 8)	300 mg AP (*n* = 5)	Total (*N* = 26)
CR	0	0	1 (12.5)	0	1 (3.8)
CRp	0	0	0	0	0
CRi/MLFS	0	0	0	1 (20.0)	1 (3.8)
Partial response	0	1 (14.3)	1 (12.5)	0	2 (7.7)
HI/SD	2 (33.3)	2 (28.6)	2 (25.0)	1 (20.0)	7 (26.9)
PD	4 (66.7)	2 (28.6)	2 (25.0)	2 (40.0)	10 (38.5)
Not evaluable/missing	0	2 (28.6)	2 (25.0)	1 (20.0)	5 (19.2)

*CR* complete remission, *CRi* complete remission with incomplete recovery of peripheral counts, *CRp* complete remission without platelet recovery, *HI* hematologic improvement, *MLFS* morphological leukemia-free state, *PD* progressive disease, *SD* stable disease

## Discussion

RO6839921 is a potent pegylated prodrug and selective new-generation antagonist of the p53-MDM2 interaction for IV administration. RO6839921 is metabolized to idasanutlin, which then binds selectively to the p53 site on the surface of the MDM2 molecule in vitro with high affinity and can effectively displace p53 from MDM2, leading to stabilization and accumulation of the p53 protein and activation of the p53 pathway. [13] In this phase 1 study in patients with AML, RO6839921 demonstrated a pharmacokinetic, pharmacodynamic, and safety profile similar to that of idasanutlin, with evidence of antileukemic activity.

RO6839921 was developed to decrease variability in exposure observed with idasanutlin and allow expansion into indications such as pediatrics or cases where patients cannot swallow or absorb the oral compound. Pediatric oral formulations of idasanutlin are in development for clinical testing (NCT04029688). The prodrug RO6839921 was cleaved rapidly to release the AP in a dose-proportional manner and demonstrated significantly improved interpatient variability compared with oral idasanutlin in solid tumors (27% vs 46%; *P* = 0.01). [17] In this study, RO6839921 also significantly improved interpatient variability of the AP compared with historical values for oral idasanutlin (33% vs 48%; *P* = 0.01), although the difference was less pronounced than in solid tumors. [18] In addition, evidence of p53 activation by RO6839921 as measured by the increase in levels of the marker MIC-1 was associated with AP exposure. [9, 16]

RO6839921 was generally well tolerated, and its safety profile was consistent with that of oral idasanutlin. [8] The most common treatment-related AEs with RO6839921 were diarrhea, nausea, vomiting, decreased appetite, and fatigue. DLTs noted at doses of 300 and 250 mg included QT interval prolongation, colitis, stomatitis, and diarrhea. By protocol definition, the MTD was 200 mg, with 0 of 7 patients having a DLT. However, in this population of patients with AML, a dose of up to 250 mg could be considered tolerable since 2 of 8 patients (25%) experienced DLT events of diarrhea and stomatitis.

Antileukemic activity (CR, CRi/MLFS, partial response, or HI/SD) was observed with RO6839921 in 11 of 26 patients, for a disease control rate of 42% and CRc rate of 8%. Other compounds in the nutlin family have also showed efficacy in patients with AML (manuscript submitted, *Lancet Haematol* August 2019). RG7112 resulted in complete remissions in a phase 1 study in patients with relapsed/refractory AML. [12] Idasanutlin has also demonstrated significant clinical activity in a phase 1 study, with some CR durations lasting >12 months in patients with relapsed/refractory AML [8]; based on these results, a phase 3 study (MIRROS; NCT02545283) is ongoing in patients with relapsed or refractory AML treated with idasanutlin in combination with cytarabine vs placebo + cytarabine. [19]

Overall, this phase 1 study showed that a soluble form of an MDM2 antagonist in the form of a pegylated prodrug to oral idasanutlin could be administered IV to patients with AML. The rapid cleavage of the prodrug RO6839921 to the AP (idasanutlin) accounts for the similar safety profile. Although RO6839921 demonstrated moderately improved variability compared with idasanutlin in patients with AML, [8] the data from this study, including those from the solid tumor arm [18], did not provide sufficient differentiation or improvement in the biologic or safety profile compared with oral idasanutlin to support continued development of the IV prodrug RO6839921.

## Data Availability

The datasets generated during and/or analyzed during the current study are available from the corresponding author on reasonable request. Qualified researchers may request access to individual patient level data through the ClinicalStudyDataRequest.com platform (www.clinicalstudydatarequest.com). Further details on Roche’s criteria for eligible studies are available here (https://clinicalstudydatarequest.com/Study-Sponsors/Study-Sponsors-Roche.aspx). For further details on Roche’s Global Policy on the Sharing of Clinical Information and how to request access to related clinical study documents, see here (https://www.roche.com/research_and_development/who_we_are_how_we_work/clinical_trials/our_commitment_to_data_sharing.htm).
